# Gender-specific prevalence and risk factors of mild cognitive impairment among older adults in Chongming, Shanghai, China

**DOI:** 10.3389/fnagi.2022.900523

**Published:** 2022-09-01

**Authors:** Yuewen Liu, Xing Yu, Peipei Han, Xiaoyu Chen, Feng Wang, Xuan Lian, Jiayu Li, Ruijin Li, Beibei Wang, Chunliu Xu, Junxue Li, Yaqing Zheng, Ziwei Zhang, Ming Li, Ying Yu, Qi Guo

**Affiliations:** ^1^Shanghai University of Medicine and Health Sciences, Shanghai, China; ^2^Shanghai University of Medicine and Health Sciences Affiliated Zhoupu Hospital, Shanghai, China; ^3^Shanghai University of Traditional Chinese Medicine, Shanghai, China; ^4^Shanghai Health Rehabilitation Hospital, Shanghai, China; ^5^Fujian Provincial Hospital, Fuzhou, China

**Keywords:** mild cognitive impairment, prevalence, risk factors, gender differences, older adults

## Abstract

**Objective:**

This study explores the gender differences in the prevalence of mild cognitive impairment (MCI) and the correlation between multiple influencing factors.

**Materials and methods:**

The sample was comprised of 1325 relatively healthy participants aged ≥ 60 years in a Shanghai community-dwelling (557 males and 768 females). Cognitive function was assessed by Mini-Mental State Examination (MMSE). The Instrumental Activities of Daily Living (IADL) scale was used to assess the activities of daily living.

**Results:**

The overall prevalence of MCI was 15.2%, with 10.2% in men and 18.9% in women. In older male subjects, those with higher the Geriatric Depression Scale (GDS) scores [odds ratio (OR) = 1.07, 95% confidence interval (CI) = 1.01–1.14] and hypertension (OR = 2.33, 95% CI = 1.15–4.73) had a higher risk of MCI. female subjects who were illiterate (OR = 2.95, 95% CI = 1.82–4.78), had a farming background (OR = 1.69, 95% CI = 1.05–2.72), and a history of stroke (OR = 1.96, 95% CI = 1.07–3.59) had a higher risk of MCI, but this was not true for males. However, Male subjects who never smoked were less likely to have MCI (OR = 0.22, 95% CI = 0.09–0.54). Additionally, the prevalence of MCI was lower in older women with high grip strength (OR = 0.96, 95% CI = 0.92–0.99) and hyperlipidemia (OR = 0.45, 95% CI = 0.22–0.96).

**Conclusion:**

The prevalence of MCI was higher in the population of elderly women compared to men. Moreover, it was found that members with MCI tended to having higher GDS scores, smoking, and hypertension; whereas a history of farming, illiteracy, stroke, grip strength, and hyperlipidemia were correlated with MCI in women.

## Introduction

China has 249 million people aged 60 and over, which accounts for 17.9% of the total population (National Bureau of Statistics of China, 2021), indicating a high prevalence of mild cognitive impairment (MCI). With the increase in the aging population worldwide, the number of patients with cognitive impairment is also increasing, putting a heavy burden on families, communities, and health care systems. Accordingly, cognitive impairment has become a global public health problem.

Due to the lack of effective treatment, early intervention is considered the most cost-effective way to manage dementia (Livingston et al., 2017). Since MCI is considered the transition stage between undamaged cognitive function and dementia (Winblad et al., 2004), there has been a consensus to focus the main intervention on this population to prevent dementia. However, a few studies investigating the prevalence of MCI in China have inconsistent results, which are estimated to range from 4.5 to 21.5% (Li et al., 2011; Song et al., 2021). These inconsistencies require further study to arrive at more accurate estimates. Although previous studies have analyzed the association between modifiable risk factors and MCI stratified by gender (Zhang et al., 2019; Fu et al., 2020), this may affect the exposure of MCI risk factors and the prevalence of MCI due to China’s vast territory, people’s complex lifestyles, extended life span, different diagnostic criteria, and various screening methods. It is estimated that more than 70% of the elderly in China live in suburban communities. Shanghai is one of the most populous megacities in China. Studying the prevalence and influencing factors of MCI in its suburban counties can provide supporting data for the prevention, diagnosis, care, and treatment of MCI. Moreover, the current research conclusions on the gender differences in the prevalence and influencing factors of MCI are still inconsistent. No gender difference in the prevalence of MCI was present in other countries (Ganguli et al., 2013; Au et al., 2017; Overton et al., 2019). The difference is that the data of China show that the prevalence of MCI in women is higher than that in men (Fu et al., 2020). Understanding the gender differences of MCI may further understand the etiology and prevention of dementia. Attention to the prevalence of MCI and effective risk factor prevention strategies will help reduce the incidence rate of MCI and subsequent dementia.

The authors’ previous studies have shown that both sarcopenia (Chen et al., 2021) and obesity (Ma et al., 2021) are associated with cognitive impairment. Therefore, this study explores the gender differences in the prevalence of MCI and the correlation between multiple influencing factors. By strengthening the identification of MCI, its related influencing factors can be examined in depth to provide a theoretical basis for the early prevention and identification of MCI to establish an early warning model of MCI.

## Materials and methods

### Study population

The final analytic sample consisted of 1,325 participants (≥ 60 years old) after excluding 38 individuals (3 had incomplete MMSE data; 4 were unable to perform the physical performance test; 31participants had missing data on covariates or outcomes.). All subjects were invited to participate in a comprehensive geriatric assessment and cognitive function assessment in Chongming District of Shanghai in 2019 and 2020. The exclusion criteria were as follows: (1) severe cognitive impairment, dementia, mental illness or other neurodegenerative diseases; (2) Inability to communicate with researchers or unwillingness to give informed consent; (3) people with deafness or blindness and cannot complete the assessment; (4) those who cannot complete the grip strength, Timed Up and Go Test (TUGT), and 4-meter walking test. All participants provided informed consent prior to participation. If the participant was illiterate, the informed consent of its legal representative would be sought.

### Covariates

Data on sociodemographic characteristics, behavioral characteristics, and disease history have previously been described (through face-to-face questions) (Liu et al., 2021). The questionnaire included questions about age, sex, height, body weight, marital status, illiteracy, living habits (alone or with others), sleep duration, smoking habits (current smoker, never smoking, and past smoker), drinking habits (drinking daily, occasional drinking, past drinking, and never drinking), and household income (< 1,000, 1,000–3,000, 3,000–5,000, and > 5,000 RMB), Physical activity was assessed using the short form of the International Physical Activity Questionnaire (IPAQ) (Jiang et al., 2009). The depressive symptoms were evaluated by the Geriatric Depression Scale (GDS). Subjects with a score of ≥ 11 were considered to have depressive symptoms (Yesavage et al., 1982). Nutrition was evaluated by the Mini Nutritional Assessment-Short Form (MNA-SF) (Kaiser et al., 2009). Physical performance was assessed by grip strength, a 4-m walking test, and the Timed Up and Go Test (TUGT). The details of the measurement method have been described in the authors’ previous research (Liu et al., 2021). Disease history included type 2 diabetes mellitus (T2DM), hypertension, hyperlipidemia, stroke, gout, anemia, pulmonary disease, biliary tract disease, kidney disease, heart disease, osteoarthritis, cancer, and thyroid disease.

### Definition of mild cognitive impairment

This study adopted the MCI diagnostic criteria based on Petersen’s definitions (Petersen et al., 1999): (1) memory complaint; (2) normal activities of daily living; (3) normal general cognitive function; (4) abnormal memory for age; (5) not demented.

### Assessment of cognitive function

Cognitive assessment was completed using the Mini-Mental State Examination (MMSE) (Folstein et al., 1975) by trained investigators. It includes 30 items, the score ranges from 0 to 30 points, with the higher scores indicating better cognitive performance. The use of MMSE to define MCI is consistent with previous studies (Liu et al., 2021), that is, The cut-off points used for cognitive impairment were as follow: ≤ 17 for illiterate people, ≤ 20 for people with primary school, and ≤ 24 for people with middle school or higher (Zhang et al., 1990). The above cut-off points have been proved to be sensitive and effective in the diagnosis of MCI in Chinese elderly population (Zhang et al., 2006).

### Assessment of daily activity ability

The daily activity ability was evaluated using the Instrumental Activities of Daily Living scale (IADL) (Lawton and Brody, 1969). It includes 8 items, the score ranges from 0 to 8 points, with the higher scores indicating better daily activity ability. IADL scores ≥ 6 indicates normal daily activity ability (Nagamatsu et al., 2013).

### Statistical analysis

The following analyses were performed to investigate the prevalence of MCI and the correlation between multiple influencing factors. Normally distributed data were presented as mean ± standard deviation, while non-normally distributed data were presented as median, with the 25–75% interquartile range given in parentheses. Categorical variables were expressed as a percentage (%). The differences according to the characteristics of cognitive status were analyzed using the *t*-tests, Chi-square tests, and Mann–Whitney *U* tests. Logistic regression analysis was used to analyze the factors associated with MCI. Based on previous research, age, marital status, illiteracy, living conditions, farming, Smoking, BMI, IPAQ, and comorbidity status (hypertension, hyperlipidemia, stroke, diabetes) were considered factors potentially associated with MCI. As such, these factors were included as independent variables in the models of the present study. All the statistical analyses were performed using SPSS version 21.0, and *P* < 0.05 was considered statistically significant.

### Ethics

The study was approved by the Ethics Committee of Shanghai University of Medicine and Health Sciences and the methods were carried out in accordance with the principles of the Declaration of Helsinki.

## Results

### Characteristics of the participants

**FIGURE 1:**
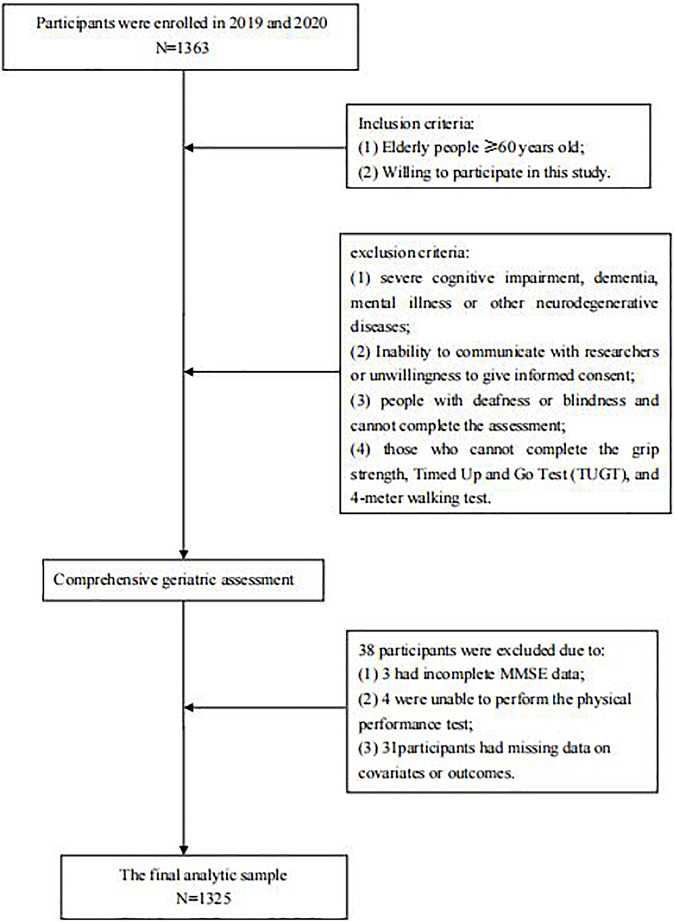
Flow chart of the study.

**FIGURE 2:**
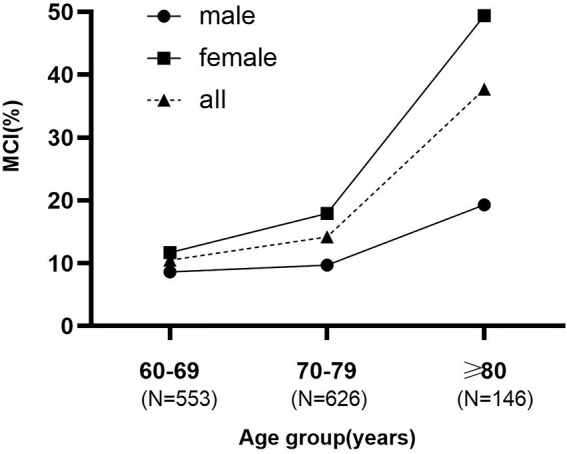
The prevalence increased more steeply with age in females than males.

A total of 1,363 participants were evaluated. Of these, 38were excluded: 3 had incomplete MMSE data; 4 were unable to perform the physical performance test; 31participants had missing data on covariates or outcomes (Figure 1). The final analytic sample consisted of 1,325 participants in the study (mean age, 71.79 ± 5.77 years; 58.0% women), of whom 202 (15.2%) were diagnosed with MCI (57 males and 145 females). Participants were divided into three age groups, 60–69, 70–79, and ≥ 80 years, with 58 (10.5%), 89 (14.2%), and 55 (37.7%) people, respectively. The prevalence increased more steeply with age in females than males (Figure 2). Table 1 presents the characteristics of these participants. Compared to participants with normal cognition, participants with MCI were older, had lower daily activity ability, higher GDS scores, sleep longer, less physical activity, and a higher proportion of illiteracy, widows, living alone, agriculture, low income, and worse physical performance. However, there were some differences when stratified by gender. Males with MCI had a higher proportion of hypertension (77.2% versus 61.8%; *P* = 0.022) and a higher score of GDS (6.54 ± 4.81 versus 5.50 ± 4.55; *P* = 0.003), but these results were not statistically significant in females (*P* > 0.05). Females with MCI had longer sleep time, lower daily activity levels, and were more likely to be farmers and illiterate.

**TABLE 1:** Baseline characteristics of study participants with normal cognition vs. mild cognitive impairment.


Variables	Total (*n* = 1325)			Male (*n* = 557)			Female (*n* = 768)		
	Normal cognition (*n* = 1123)	Mild cognitive impairment (*n* = 202)	*P-value*	Normal cognition (*n* = 500)	Mild cognitive impairment (*n* = 57)	*P-value*	Normal cognition (*n* = 623)	Mild cognitive impairment (*n* = 145)	*P-value*
Age (year)	71.27 ± 5.38	74.66 ± 6.96	<0.001	71.70 ± 5.48	73.72 ± 7.02	0.001	70.93 ± 5.28	75.03 ± 6.92	<0.001
BMI(kg/m^2^)	23.69 ± 3.45	23.68 ± 3.43	0.963	23.40 ± 3.33	23.39 ± 3.83	0.989	23.92 ± 3.53	23.79 ± 3.27	0.680
IADL (score)	7.79 ± 0.63	7,63 ± 0.67	0.001	7.82 ± 0.47	7.68 ± 0.61	0.039	7.77 ± 0.73	7.61 ± 0.69	0.021
MNA-SF (score)	12.79 ± 1.51	12.68 ± 1.44	0.327	12.82 ± 1.44	12.51 ± 1.66	0.127	12.76 ± 1.56	12.74 ± 1.34	0.886
GDS (score)	5.50 ± 4.55	6.81 ± 5.39	<0.001	4.85 ± 4.01	6.54 ± 4.81	0.003	6.02 ± 4.89	6.92 ± 5.61	0.053
Sleep duration(hour)	8.40 ± 1.43	8.79 ± 1.55	0.001	8.43 ± 1.39	8.61 ± 1.49	0.334	8.38 ± 1.45	8.85 ± 1.57	0.001
IPAQ (Met/wk)	5255(2226,10080)	3864(1647,8201)	0.003	4988(2079, 8966)	3222(1533, 8652)	0.167	5639(2373, 10920)	3990(1908, 7989)	0.003
Illiteracy (%)	98(8.7%)	68(33.7%)	<0.001	21(4.2%)	5(8.8%)	0.121	77(12.4%)	63(43.4%)	<0.001
Widowed(%)	191(17.0%)	75(37.1%)	<0.001	41(8.2%)	11(19.3%)	0.006	150(24.1%)	64(44.1%)	<0.001
Living alone (%)	166(14.8%)	61(30.2%)	<0.001	50(10.0%)	14(24.6%)	0.001	116(18.6%)	47(32.4%)	<0.001
Farming (%)	565(50.3%)	135(66.8%)	<0.001	198(39.6%)	23(40.4%)	0.913	367(58.9%)	112(77.2%)	<0.001
**Monthly income (%)**			<0.001			0.121			0.004
< 1000	70(6.2%)	29(14.4%)		23(4.6%)	6(10.5%)		47(7.5%)	23(15.9%)	
1000-3000	683(60.8%)	131(64.9%)		286(57.2%)	36(63.2%)		397(63.7%)	95(65.5%)	
3000-5000	160(14.2%)	18(8.9%)		80(16.0%)	7(12.3%)		80(12.8%)	11(7.6%)	
>5000	210(18.7%)	24(11.9%)		111(22.2%)	8(14.0%)		99(15.9%)	16(11.0%)	
**Smoking (%)**			0.014			0.075			0.495
Current smokers	178(15.9%)	28(13.9%)		174(34.8%)	28(49.1%)		4(0.6%)	0(0.0%)	
Never smokers	757(67.4%)	155(76.7%)		140(28.0%)	10(17.5%)		617(99.0%)	145(100%)	
Ever smokers	188(16.7%)	19(9.4%)		186(37.2%)	19(33.3%)		2(0.3%)	0(0.0%)	
**Drinking (%)**			0.065			0.168			0.126
Daily drinkers	171(15.2%)	29(14.4%)		150(30.0%)	18(31.6%)		21(3.4%)	11(7.6%)	
Occasional drinkers	152(13.5%)	27(13.4%)		91(18.2%)	16(28.1%)		61(9.8%)	11(7.6%)	
Former drinkers	135(12.0%)	12(5.9%)		111(22.2%)	7(12.3%)		24(3.9%)	5(3.4%)	
Never drinkers	665(59.2%)	134(66.3%)		148(29.6%)	16(28.1%)		517(83.0%)	118(81.4%)	
**physical performance**									
Grip strength (kg)	23.95 ± 8.91	19.10 ± 8.88	<0.001	30.31 ± 8.22	27.01 ± 9.65	0.005	18.85 ± 5.50	15.99 ± 6.28	<0.001
4-meter walking test (m/s)	1.11 ± 0.23	0.94 ± 0.25	<0.001	1.15 ± 0.25	1.02 ± 0.25	0.001	1.07 ± 0.21	0.92 ± 0.25	<0.001
TUGT (s)	9.63 ± 3.31	11.9 ± 4.76	<0.001	9.56 ± 3.36	11.04 ± 3.70	0.002	9.68 ± 3.28	12.24 ± 5.09	<0.001
**Diseases (%)**									
Diabetes (%)	178(15.9%)	32(15.8%)	0.997	78(15.6%)	9(15.8%)	0.970	100(16.1%)	23(15.9%)	0.955
Hypertension (%)	683(60.8%)	139(68.8%)	0.031	309(61.8%)	44(77.2%)	0.022	374(60.0%)	95(65.5%)	0.222
Hyperlipidemia (%)	140(12.5%)	17(8.4%)	0.100	56(11.2%)	7(12.3%)	0.811	84(13.5%)	10(6.9%)	0.029
Stroke(%)	114(10.2%)	29(14.4%)	0.076	51(10.2%)	5(8.8%)	0.734	63(10.1%)	24(16.6%)	0.028
Gout (%)	66(5.9%)	10(5.0%)	0.613	43(8.6%)	5(8.9%)	0.934	23(3.7%)	5(3.4%)	0.888
Anemia (%)	51(4.5%)	13(6.4%)	0.248	18(3.6%)	3(5.3%)	0.532	33(5.3%)	10(6.9%)	0.450
Pulmonary disease (%)	96(8.5%)	15(7.4%)	0.596	58(11.6%)	6(10.5%)	0.810	38(6.1%)	9(6.2%)	0.961
Biliary tract disease (%)	166(14.8%)	32(15.8%)	0.697	49(9.8%)	7(12.3%)	0.555	117(18.8%)	25(17.2%)	0.667
Kidney disease (%)	117(10.4%)	12(5.9%)	0.048	70(14.0%)	5(8.8%)	0.273	47(7.5%)	7(4.8%)	0.249
Heart disease (%)	359(32.0%)	73(36.1%)	0.244	142(28.4%)	19(33.3%)	0.436	217(34.8%)	54(37.2%)	0.584
Thyroid disease (%)	44(3.9%)	10(5.0%)	0.494	11(2.2%)	0(0.0%)	0.258	33(5.3%)	10(6.9%)	0.450
Osteoarthritis(%)	179(15.9%)	39(19.3%)	0.235	68(13.6%)	11(19.3%)	0.243	111(17.8%)	28(19.3%)	0.674
Cancer(%)	44(3.9%)	6(3.0%)	0.515	26(86.7%)	4(7.0%)	0565	18(2.9%)	2(1.4%)	0.304

BMI, Body Mass Index; MNA, Mini Nutritional Assessment; TUGT, Time Up and Go Test; GDS, Geriatric Depression Scale; IADL, Instrumental Activity of Daily Living; IPAQ, International Physical Activity Questionnaires.

### Univariate and multivariate analysis of associated factors for mild cognitive impairment

Results from univariate and multivariate logistic regression models for factors related to MCI in 557 males and 768 females are reported in Tables 2 and 3, respectively. After adjusting for potential confounders, in older male subjects, those with higher GDS scores [odds ratio (OR) = 1.07, 95% confidence interval (CI) = 1.01–1.14] and hypertension (OR = 2.33, 95% CI = 1.15–4.73) were associated with MCI. Male subjects who never smoked were less likely to have MCI than older male subjects who currently smoked (OR = 0.22, 95% CI = 0.09–0.54). Female subjects who were illiterate (OR = 2.95, 95% CI = 1.82–4.78), had a background in farming (OR = 1.69, 95% CI = 1.05–2.72), and had high grip strength (OR = 0.96, 95% CI = 0.92–0.99) were associated with MCI, but this was not true for males. The OR and 95% CI in the adjusted model for the factors statistically significantly associated with MCI were 0.45 (0.22–0.96) and 1.96 (1.07–3.59) for hyperlipidemia and stroke, respectively, in females.

**TABLE 2:** Unadjusted and Adjusted Model for Factors Related to MCI in the male.

Variable	Univariate Odds Ratio (95% CI)	*P-value*	Adjusted Model Odds Ratio (95% CI)	*P-value*
All sample (*n* = 557)				
Age (year)				
60-69 (*n* = 221)	1.0 (referent)		1.0 (referent)	
70-79 (*n* = 279)	1.14(0.62–2.11)	0.678	1.08(0.54–2.15)	0.823
≥ 80 (*n* = 57)	2.54(1.13–5.71)	0.024	1.44(0.49–4.27)	0.507
IADL	0.63(0.40–0.99)	0.044	0.83(0.47–1.44)	0.501
GDS	1.09(1.03–1.15)	0.004	1.07(1.01–1.14)	0.033
Illiteracy (*n* = 26)	2.19(0.79–6.06)	0.130	1.74(0.53–5.69)	0.358
Widowed (*n* = 52)	2.68(1.29–5.56)	0.008	1.15(0.33–4.01)	0.825
Living alone (*n* = 64)	2.93(1.50–5.73)	0.002	1.86(0.64–5.36)	0.252
Farming (*n* = 221)	1.03(0.59–1.80)	0.913	0.70(0.36–1.35)	0.283
**Monthly income**				
< 1,000 (*n* = 29)	1.0 (referent)		1.0 (referent)	
1,000–2,999 (*n* = 322)	0.48(0.18–1.26)	0.138	0.44(0.14–1.36)	0.152
3,000–5,000 (*n* = 87)	0.34(0.10–1.10)	0.071	0.29(0.07–1.16)	0.080
>5,000 (*n* = 119)	0.28(0.09–0.87)	0.028	0.28(0.07–1.12)	0.071
**Smoking**				
Current smokers (*n* = 202)	1.0 (referent)			
Never smokers (*n* = 150)	0.44(0.21–0.95)	0.035	0.22(0.09–0.54)	0.001
Ever smokers (*n* = 205)	0.64(0.34–1.18)	0.150	0.47(0.24–0.95)	0.036
Grip strength	0.95(0.92–0.99)	0.005	0.97(0.93–1.01)	0.105
4-meter walking test	0.16(0.05–0.46)	0.001	0.23(0.04–1.23)	0.085
TUGT	1.10(1.03-1.17)	0.005	0.98(0.88–1.09)	0.670
Hypertension (*n* = 353)	2.09(1.10–3.99)	0.025	2.33(1.15–4.73)	0.020
Hyperlipidemia (*n* = 63)	1.11(0.48–2.56)	0.811	1.08(0.42–2.79)	0.879
Stroke (*n* = 56)	0.85(0.32–2.22)	0.734	0.72(0.25–2.12)	0.554

TUGT, time up and go; OR, Odds Ratio; CI, confidence interval; IPAQ, international physical activity questionnaires; Adjusted model: Adjusted for age, BMI, IADL, GDS, IPAQ, Illiteracy, Widowed, Living alone, Farming, Monthly income, Smoking, Grip strength, 4-meter walking test, TUGT, Hypertension, Hyperlipidemia, Stroke, diabetes.

**TABLE 3:** Unadjusted and adjusted model for factors related to MCI in the female.

Variable	Univariate Odds Ratio (95% CI)	*P-value*	Adjusted Model Odds Ratio (95% CI)	*P-value*
All sample (*n* = 768)				
Age (year)				
60-69 (*n* = 332)	1.0 (referent)		1.0 (referent)	
70-79 (*n* = 347)	1.64(1.06–2.52)	0.026	1.02(0.62–1.67)	0.939
≥ 80 (*n* = 89)	7.35(4.31–12.52)	<0.001	1.81(0.90–3.63)	0.097
IADL	0.79(0.63–0.98)	0.028	1.26(0.93–1.72)	0.142
GDS	1.03(0.99–1.07)	0.054	0.99(0.95–1.03)	0.608
Sleep duration	1.22(1.08–1.38)	0.001	1.05(0.92–1.20)	0.456
IPAQ	0.96(0.92–0.99)	0.001	0.98(0.96–1.09)	0.131
Illiteracy (*n* = 140)	5.45(3.63–8.18)	<0.001	2.95(1.82–4.78)	<0.001
Widowed (*n* = 214)	2.49(1.71–3.63)	<0.001	1.50(0.78–2.88)	0.226
Living alone (*n* = 163)	2.10(1.40–3.13)	<0.001	0.85(0.41–1.72)	0.642
Farming (*n* = 479)	2.37(1.56–3.60)	<0.001	1.69(1.05–2.72)	0.031
**Monthly income**				
< 1,000 (*n* = 70)	1.0 (referent)		1.0 (referent)	
1,000-2,999 (*n* = 492)	0.49(0.28–0.85)	0.010	0.67(0.35–1.30)	0.235
3,000-5,000 (*n* = 91)	0.28(0.13–0.63)	0.002	0.53(0.21–1.34)	0.179
>5,000 (*n* = 115)	0.33(0.16–0.68)	0.003	0.65(0.28–1.55)	0.330
Grip strength	0.91(0.88–0.94)	<0.001	0.96(0.92–0.99)	0.036
4-meter walking test	0.06(0.03–0.13)	<0.001	0.40(0.11–1.42)	0.156
TUGT	1.16(1.11–1.22)	<0.001	1.05(0.98–1.12)	0.142
Hypertension (*n* = 469)	1.27(0.87–1.85)	0.223	1.15(0.74–1.81)	0.537
Hyperlipidemia (*n* = 94)	0.48(0.24–0.94)	0.033	0.45(0.22–0.96)	0.038
Stroke (*n* = 87)	1.76(1.06–2.94)	0.029	1.96(1.07–3.59)	0.030

TUGT, time up and go; OR, Odds Ratio; CI, confidence interval; IPAQ, international physical activity questionnaires; Adjusted model: Adjusted for age, BMI, IADL, GDS, Sleep duration, IPAQ, Illiteracy, Widowed, Living alone, Farming, Monthly income, Smoking, Grip strength, 4-meter walking test, TUGT, Hypertension, Hyperlipidemia, Stroke, diabetes.

## Discussion

This study estimates the prevalence of MCI and the factors associated with MCI in a suburb-dwelling population of elderly persons aged 60 and older in China. The overall prevalence of MCI was 15.2%, with 10.2% in men and 18.9% in women. After adjustment for potential confounders, the GDS scores, smoking, and hypertension were directly associated with the prevalence of MCI in men. Moreover, grip strength, being illiterate, having a background in farming, hyperlipidemia, and stroke were associated with the prevalence of MCI in women.

### Prevalence of mild cognitive impairment in suburb-dwelling residents

A recent meta-analysis showed that the prevalence of MCI in the Chinese elderly was 15.2% (Deng et al., 2021), which was consistent with the results of the present study. A recent cross-sectional survey on the prevalence of MCI in women showed that the prevalence of MCI was 21.5% (Song et al., 2021), while another study found that the prevalence of cognitive impairment was 26.1% in men and 30.5% in women (Lyu and Kim, 2016). Additionally, a cross-sectional study found that the prevalence of MCI was 19.8% for men and 26.1% for women (Zhang et al., 2019). In the study by Fu et al. (2020), the MCI prevalence was 8.2% in males and 13.1% in females. These large differences in prevalence are likely due to diversity in population structure, screening tools, and diagnostic criteria. These inconsistencies necessitate further study to yield a more accurate estimate.

### Modifiable lifestyle and physical performance factors related to mild cognitive impairment in suburb-dwelling residents

The present study found that GDS scores were correlated with the prevalence of MCI. This was consistent with the results of previous studies (Vega and Newhouse, 2014). There is increasing evidence that depression strongly and independently accelerates the progression from MCI to dementia. In individuals with MCI, depressive symptoms mean a 2–4 fold increase in the risk of progression to dementia (Van der Mussele et al., 2014). In men, an association was found between smoking and MCI. This result was consistent with previous work that reported smoking was associated with cognitive impairment (Jia et al., 2020). Regarding the relationship between smoking and MCI, further studies on biomarker assessments of smoking are needed to illustrate the true association between smoking and MCI in the Chinese population. This result also showed that MCI was significantly associated with a background in farming and illiteracy in women. A systematic review showed that those who have a predominantly manual occupation throughout life have a greater risk of cognitive impairment and/or dementia than those in intellectually demanding occupations (Gracia et al., 2016). Illiterate women were more than twice as likely to have MCI than educated women. Previous studies have found that women with little formal education have a higher risk of dementia than men with similar educational backgrounds (Zhang et al., 1990; Launer et al., 1999; Ott et al., 1999). Differences in the quality of education may contribute to an increased risk of dementia in women compared with men. Low-educated women are more likely to have poorer career achievement, lower income, poorer health, less leisure opportunities, and poorer cognitive outcomes than low-educated men (Ott et al., 1999; Sharp and Gatz, 2011). Compared with men, women have less access to education, and when women have access to education, it differs in quality. Zhang et al. (2006) point out that before 1950, only women belonging to the highest social classes could receive education in China. Additionally, a relationship was found between MCI and grip strength in women, as was found in previous studies (Atkinson et al., 2010). This simple measure may be useful for future research on the relationship between cognitive function and physical performance.

### Specific diseases associated with mild cognitive impairment in suburb-dwelling residents

As demonstrated in the present study, hypertension was associated with MCI in men, but not in women. This finding was in accordance with previous studies (Wu et al., 2016; Fu et al., 2020). The potential gender differences between hypertension and cognition are not fully understood (Iadecola et al., 2016). It may be that the women in the present study were all menopausal. Previous evidence suggests that older postmenopausal women have a lower risk of cardiometabolic disease because of their favorable hormonal and metabolic profile (Regensteiner et al., 2015), which may mitigate cognitive impairment caused by hypertension. However, the observational studies conducted for the present study could not provide insight into the underlying biological mechanisms. Accordingly, more research should be conducted to explore possible explanations for the underlying gender differences. In addition, similar to previous studies (Kim and Park, 2017; Fu et al., 2020), the present study found no significant correlation between hyperlipidemia and the prevalence of MCI in the elderly. Interestingly, when stratified by gender, the association between hyperlipidemia and MCI was only significant in women, and women with hyperlipidemia had higher cognitive function than women without hyperlipidemia. In the study conducted by Kim and Park (2017), hyperlipidemia was reported as a protective factor for MCI in women. The relationship between hyperlipidemia and MCI prevalence remains controversial. Studies have shown that a reduced risk of cognitive impairment was associated with higher serum cholesterol levels (Lv et al., 2016). In contrast, earlier research found significantly higher cholesterol levels in older adults with cognitive impairment and suggested that cholesterol-lowering treatments, such as lipid-lowering drugs, may have cardiovascular benefits while preventing cognitive impairment (Vance, 2012). However, the underlying mechanism of the interaction between gender and hyperlipidemia and cognitive impairment remains unclear. Further prospective studies are needed to explore the sex differences with the association of hyperlipidemia with MCI. A recent meta-analysis showed that stroke was a risk factor for dementia, which was consistent with the results of the present study (Deng et al., 2021). Similarly, a study examining gender differences in cognitive outcomes after stroke found that women had significantly worse cognitive outcomes than men (Dong et al., 2020). Since only a few previous works investigated the gender differences between stroke and MCI, further research is needed to explore the potential pathological associations.

### Strengths and limitations

This study presents several strengths. First, the study was conducted on a relatively large sample of well-characterized suburban elderly men and women who had lived in a particular geographic area for a long time. Second, the participants were from suburban areas, and their lifestyles were more active, which may have been different from subjects in other areas. However, this study has some limitations that must be addressed. First, because this is a cross-sectional study, a causal relationship of the identified associated factors could not be established. Second, all participants in this study were relatively healthy. We did not include those who were hospitalized, or those who were bedridden with severe illness and could not be tested on-site, and those who refused to participate in the program. This choice could constitute a selective survival and a healthy selection bias. Third, although there were no patients with depression in our population, we found that men with higher GDS scores had worse cognitive function, which we also adjusted as an adjustment variable in multivariate regression. In the future, we will continue to follow up to observe the impact of depression on cognitive function.

## Conclusion

In summary, in this study, 15.2% of adults aged 60 and older were found to have mild cognitive impairment. The prevalence of MCI is shown to be higher in females compared to males for the elderly population. Furthermore, GDS scores, smoking, farming, illiteracy, grip strength, hypertension, hyperlipidemia, and stroke may have different associations with MCI in different genders. Additional studies with longer follow-up periods are needed to confirm these associations and to further examine other lifestyle behaviors and diseases that may contribute to MCI. This study may be of great significance to the prevention of MCI and ensuring a healthy aging policy.

## Data availability statement

The original contributions presented in this study are included in the article/supplementary material, further inquiries can be directed to the corresponding author/s.

## Ethics statement

The studies involving human participants were reviewed and approved by the Ethics Committee of Shanghai University of Medicine and Health Sciences. The patients/participants provided their written informed consent to participate in this study.

## Author contributions

YY, QG, and ML contributed to the conception and design of the study. YL wrote the first draft of the manuscript. PH organized the database. XY performed the statistical analysis. XC, FW, JuL, YZ, and ZZ wrote sections of the manuscript. XL, JiL, RL, BW, and CX participate in the survey. All authors contributed to the article and approved the submitted version.
