# Erratum: Gender-specific prevalence and risk factors of mild cognitive impairment among older adults in Chongming, Shanghai, China

**DOI:** 10.3389/fnagi.2022.1085622

**Published:** 2022-11-09

**Authors:** Frontiers Production Office

**Affiliations:** Frontiers Media SA, Lausanne, Switzerland

**Keywords:** mild cognitive impairment, prevalence, risk factors, gender differences, older adults

Due to a production error, there was a mistake in Figures 1, 2 as published. The order of these figures was reversed. The corrected Figures 1, 2 appear below.

**Figure 1:**
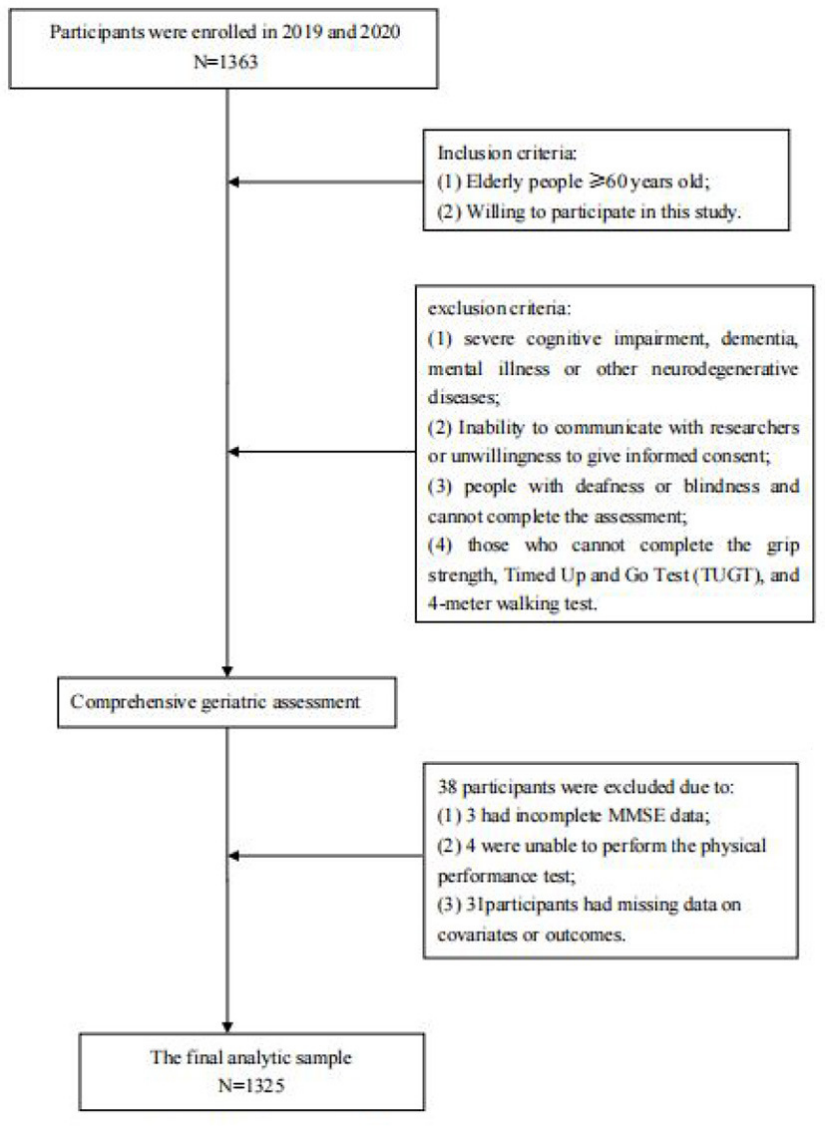
Flow chart of the study.

**Figure 2:**
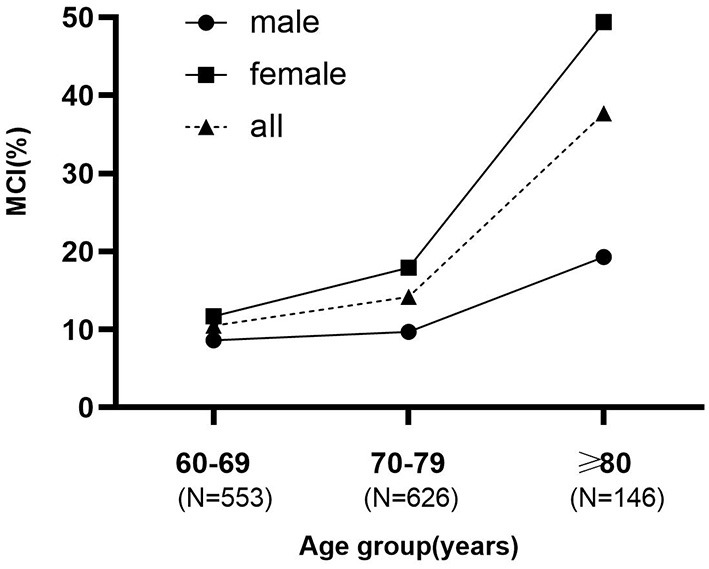
The prevalence increased more steeply with age in females than males.

The publisher apologizes for this mistake. The original version of this article has been updated.

